# Laboratory experiment of ATP measurement using Mars soil simulant: as a method for extraterrestrial life detection

**DOI:** 10.1007/s44211-022-00081-y

**Published:** 2022-02-25

**Authors:** Keigo Enya, Satoshi Sasaki

**Affiliations:** 1 Institute of Space and Astronautical Science, Japan Aerospace Exploration Agency, 3-1-1 Yoshinodai, Chuo, Sagamihara, Kanagawa 252-5210 Japan; 2 The Graduate University for Advanced Studies, SOKENDAI, Hayama, Miura, Kanagawa 240-0193 Japan; 3 School of Health Sciences/Bioscience and Biotechnology, Tokyo University of Technology, 5-23-22 Nishikamata, Ohta, Tokyo 144-8535 Japan

**Keywords:** ATP, Mars simulant, *Escherichia coli*, Experiment, Extraterrestrial life

## Abstract

We present a laboratory experiment of ATP measurement using Mars soil simulant and *Escherichia coli* (*E. coli*) with a point of view for future application to searching extraterrestrial life. We used Mars Global Simulant MGS-1 (Exolith Lab) as soil simulant, added *E. coli* suspension to it, then the soil simulant with *E. coli* was dried. Various configurations of samples with different *E. coli* density, 1.75 × 10^2^, 1.75 × 10^3^, 1.75 × 10^4^, 1.75 × 10^5^, and 1.75 × 10^6^ cells (g soil)^−1^, were prepared together with controls. ATP extraction reagent and luminescence reagent were added to the sample, and bioluminescence measurement was performed. The result suggests significant detection of ATP for samples with *E. coli* density used in this work. Similar experiments but without the soil simulant were carried out, and results with and without the soil simulant are compared. Based on the ATP measurement studied in this work, we discussed extraterrestrial life search, planetary protection, relation with the panspermia hypothesis, and also other applications.

**Graphical abstract:**

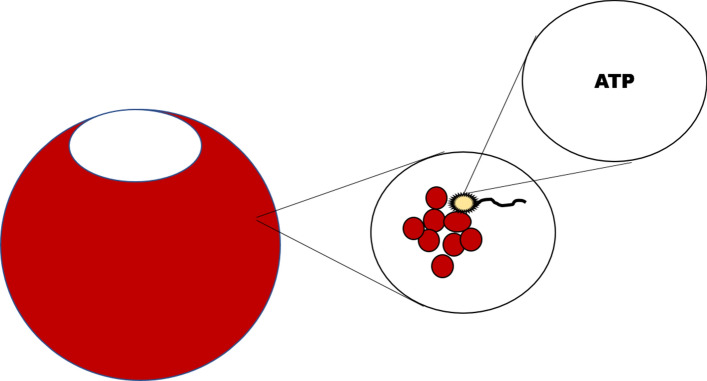

## Introduction

We consider that detection and analysis of extraterrestrial life is one of the most important issue of space science in this century. Thus, various methods are considered for these aims. It is one of the reasonable approach to search extraterrestrial life based on features of terrestrial life (while on the other hand we should not forget possibility features of extraterrestrial life are completely different from those of terrestrial life).

Adenosine triphosphate (ATP) is equipped inside of all of the known terrestrial lifeform to use energy. Strehler and Totter [1] reported a method to detect adenosine triphosphate (ATP) using optical measurement of bioluminescence. In this method, ATP is led to bioluminescence by the action of enzymes [2]. Bioluminescence measurement provides high sensitivity. So ATP measurement via bioluminescence is widely applied to hygiene management because it can measure ATP contained in microorganisms and organic matter derived and food residues [3]. ATP measurement in the natural environment on the earth was also reported [4, 5]. Applicability of ATP measurement to extraterrestrial life search was studied [6].

Mars is considered as the most important place for extraterrestrial life search because of the past and current environment on Mars. The Viking mission of NASA executed pioneering experiments for life detection on Mars [7–10]. The Viking experiments could not conclude detection of life because of the limit of sensitivity. Afterwards, a Mars rover of NASA, Curiosity, detected organic matter on Mars [11–13]. However, organic matter exists in meteorites for example. So it is unknown if the organic matter detected by Curiosity was produced by life. There is no liquid open water on current Mars. Therefore, it is reasonable to use soil samples for life search on Mars as Viking mission did.

It is not guaranteed that extraterrestrial life uses ATP. However, based on universality of using ATP among terrestrial life, we consider that it is also possible that extraterrestrial life has been evolved to use ATP. There is a possibility to find ATP produced by a past life. Another possible scenario is that an ancestor moved interplanetary [14, 15]. In this case, it is more reasonable that the extraterrestrial life uses ATP to keep characteristics of its ancestor and therefore uses ATP. Measurement of ATP is important to examine this hypothesis even if the result is negative (i.e., no detection of ATP).

Moreover, ATP measurement is also important for the planetary protection. ATP measurement provides a high sensitivity test to evaluate if a spacecraft, an instrument onboard it, and planetary surface around the landed place of a spacecraft are contaminated by terrestrial life.

**Fig. 1:**
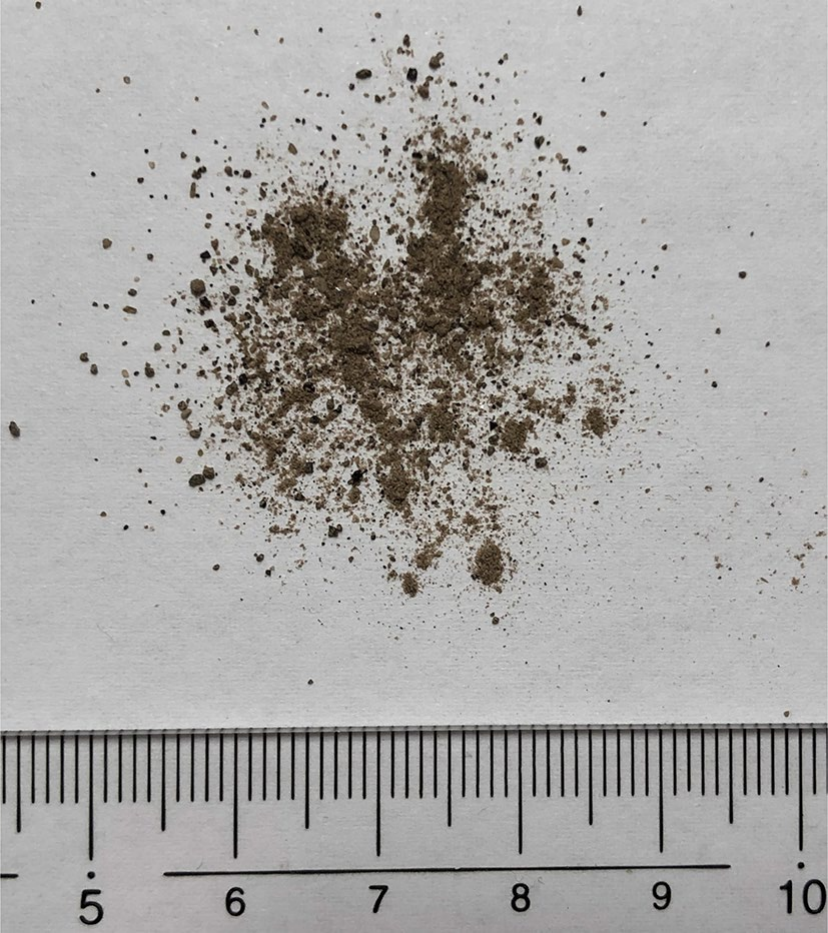
A photograph of the Mars soil simulant. The unit of number on the scale is cm

Considering these situations, in this work, we prepared samples by adding *Escherichia coli* (*E. coli*) to the soil simulant on Mars (Fig. 1) with various bacterial densities, and conducted an experiment to detect ATP. Because an instrument to be carried to Mars have to be compact, light weighted, and simple, we applied ATP measurement by bioluminescence directly to the mixed soil simulant and *E. coli* (i.e., without procedure to separate the soil).

## Materials and methods

As summarized in Table 1, we prepared samples consisting of 13 configurations. Ecoli-1s, Ecoli-2s, Ecoli-3s, Ecoli-4s, and Ecoli-5s are configuration including the Mars soil simulant and various densities of *E. coli*. Ecoli-1n, Ecoli-2n, Ecoli-3n, Ecoli-4n, and Ecoli-5n are also configuration including the various density of *E. coli*., but with no soil simulant. Tube, Ecoli-0s, and Ecoli-0n are prepared as controls. Four samples were prepared for each configuration.

**Table 1:** Configuration of samples

Configuration name	Quantity of sample	Test tube	Soil simulant /mg	*E. coli* /cells (g soil)^−1^	Extraction reagent /μL	Luminescence reagent /μL
Tube	4 (#1, #2, #3, #4)	Yes	0	0	0	0
Ecoli-0s	4 (#1, #2, #3, #4)	Yes	10	0	100	100
Ecoli-1s	4 (#1, #2, #3, #4)	Yes	10	1.75 × 10^2^	100	100
Ecoli-2s	4 (#1, #2, #3, #4)	Yes	10	1.75 × 10^3^	100	100
Ecoli-3s	4 (#1, #2, #3, #4)	Yes	10	1.75 × 10^4^	100	100
Ecoli-4s	4 (#1, #2, #3, #4)	Yes	10	1.75 × 10^5^	100	100
Ecoli-5s	4 (#1, #2, #3, #4)	Yes	10	1.75 × 10^6^	100	100
Ecoli-0n	4 (#1, #2, #3, #4)	Yes	0	0	100	100
Ecoli-1n	4 (#1, #2, #3, #4)	Yes	0	1.75 × 10^2^	100	100
Ecoli-2n	4 (#1, #2, #3, #4)	Yes	0	1.75 × 10^3^	100	100
Ecoli-3n	4 (#1, #2, #3, #4)	Yes	0	1.75 × 10^4^	100	100
Ecoli-4n	4 (#1, #2, #3, #4)	Yes	0	1.75 × 10^5^	100	100
Ecoli-5n	4 (#1, #2, #3, #4)	Yes	0	1.75 × 10^6^	100	100

**Fig. 2:**
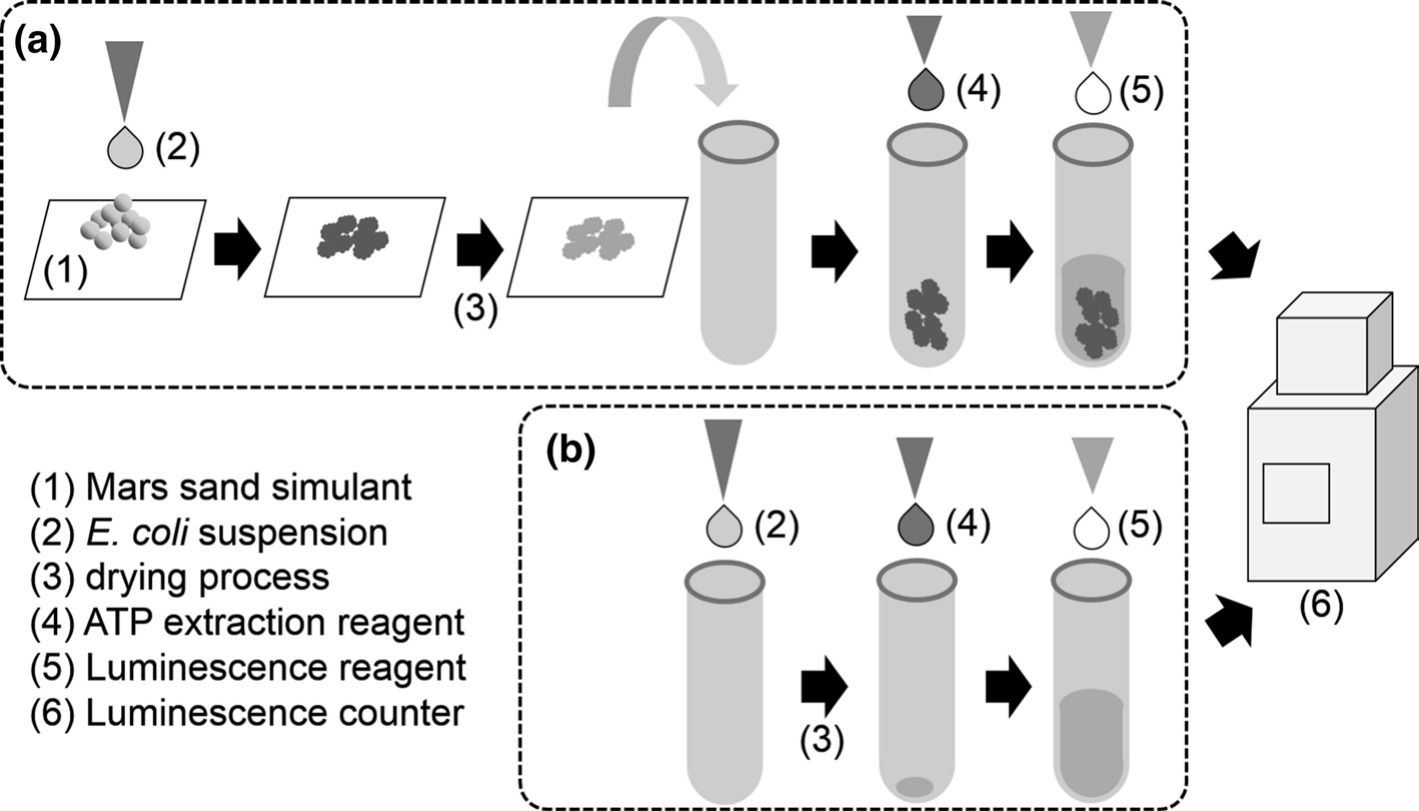
Process for sample preparation and measurement. **a** and **b** Are for process with and without the Mars soil simulant, respectively

Procedures of sample preparation and measurement are illustrated in Fig. 2. A kit for bioluminescence-based ATP measurement was used (ATP Assay Kit Type LL100-1-2, TOYO BNet Co., Ltd, Tokyo, Japan), where ATP and D-luciferin react through the catalysis of luciferase to produce light, oxyluciferin, diphosphate, adenosine monophosphate (AMP) and CO_2_ as products. Extraction reagent (ATP extraction reagent Type LL100-2, TOYO BNet Co., Ltd, Tokyo, Japan) was used for the extraction of ATP from the *E. coli*. A photomultiplier-based luminescence detector (LUMICOUNTER 2500, Microtec Co., Ltd.) was used for the measurement, followed by data transmission/recording using a terminal emulator application on a computer. A model microorganism, *E. coli*, was cultured overnight at 37 °C from a glycerol stock using Luria-Berterni (LB) broth, followed by washing using phosphate buffer solution (PBS (-) w/o Ca^2+^, Mg^2+^, Cell Science & Technology Institute Inc.) for 3 times by centrifuge (8000 G, 30 min). Cell density of the PBS suspension was estimated based on colony-forming unit using LB agar plate.

For the experiment with samples including *E. col*i and the Mars soil simulant, 3 μL of the *E. coli* suspension was dropped onto 10 mg of the soil simulant (Mars Global Simulant MGS-1, Exolith Lab), followed by air drying for 5 min. in a clean bench. Dried soil simulant with *E. coli* was placed in a test tube and then 100 μL of ATP extraction reagent was added. About 20 min later, 100 μL of luminescence reagent was added into the test tube and mixed. Then the tube was immediately placed inside the measurement chamber of the luminescence counter. Luminescence intensity was measured for 60 s with 1 s interval. For configurations that do not include the soil simulant, the same procedure but without the soil simulant was applied.

## Results

**Fig. 3:**
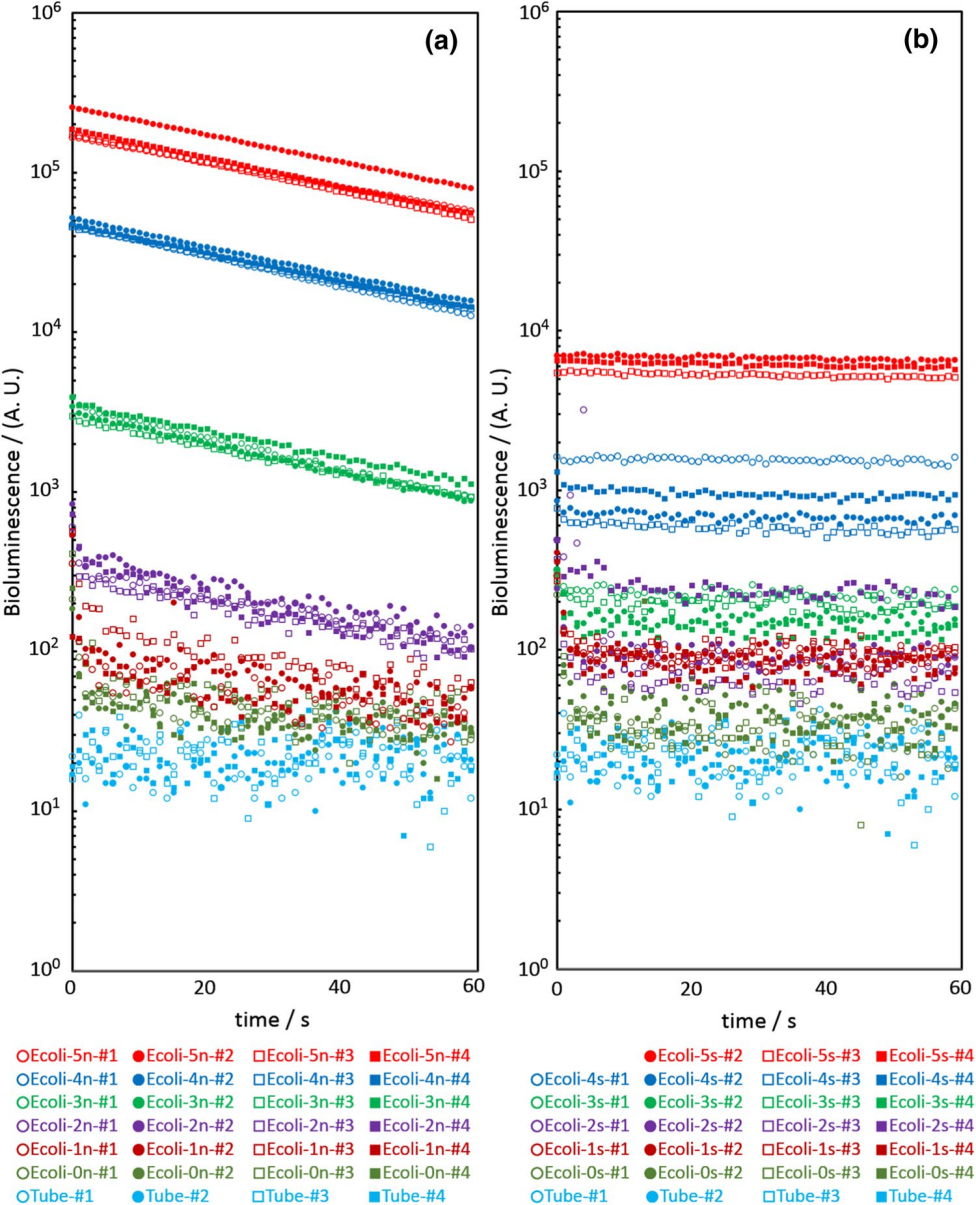
Bioluminescence data obtained by 1 min measurement for each samples. **a** Shows data for configuration without the Mars soil simulant. Data for configuration of Tube is also shown for comparison. **b** Shows similar data but for configuration with the soil simulant. A. U. is an abbreviation for arbitrary unit

Data of bioluminescence obtained by 1 min measurement for each sample is shown in Fig. 3. Table 2 shows a summary of Average and standard deviation of measured bioluminescence. It should be noted that arbitrary unit (A. U.) is used for bioluminescence output from the photomultiplier in Fig. 3 and Table 2.

**Table 2:** Average and standard deviation of measured bioluminescence

Configuration name	(1) Sample #1 average /(A. U.)*	(2) Sample #2 average /(A. U.)	(3) Sample #3 average /(A. U.)	(4) Sample #4 average /(A. U.)	(5) Average of (1)–(4) /(A. U.)	(6) σ of (1)–(4) /(A. U.)
Tube	22.1	20.9	22.8	21.9	21.9	0.7
Ecoli-0s	46.7	43.2	56.1	42.0	47.0	5.5
Ecoli-1s	94.1	97.3	103.1	83.9	94.6	7.0
Ecoli-2s	168.3	96.5	70.1	245.3	145.0	78.7
Ecoli-3s	225.0	163.3	197.5	137.0	180.7	38.5
Ecoli-4s	1540.0	685.3	586.0	954.3	941.4	428.3
Ecoli-5s	–	6752.1	5293.7	6151.7	6065.8	733.0
Ecoli-0n	35.3	48.3	38.3	42.1	41.0	5.6
Ecoli-1n	59.9	83.1	104.5	58.5	76.5	21.8
Ecoli-2n	201.6	244.1	181.6	202.6	207.5	26.2
Ecoli-3n	1904.4	1762.9	1696.5	2120.7	1871.1	187.6
Ecoli-4n	25,968.0	29,858.2	26,655.7	27,455.2	27,484.3	1695.3
Ecoli-5n	102,619.8	150,776.0	100,422.0	107,814.0	115,408.0	23,781.5

(1)–(4): Average of data of the 1 min measurement for each sample. A. U. is an abbreviation for arbitrary unit

(5): Average of (1)–(4). For Ecoli-5s, average of (2)–(4)

(6): Standard deviation of (1)–(4). For Ecoli-5s, standard deviation of (2)–(4)

In the data of the 1 min measurement for samples including the Mars soil simulant (i.e., Ecoli-0s−Ecoli-5s), positive correlation between the density of *E. coli*. and the bioluminescence is found, and each data of the 1 min measurement is almost constant. In the data of the 1 min measurement for samples with no soil simulant (i.e., Ecoli-0n–Ecoli-5n), positive correlation between the density of *E. coli* and the bioluminescence is found, as recognized for the samples including the soil simulant. However, the bioluminescence decreases exponentially in the 1 min measurement. It is also found that the intensity of the bioluminescence of the sample with no soil remnant is clearly higher than the sample with the soil remnant.

In an experiment for Ecoli-5s, we failed handling of one sample. As the result, there are only three measurement data for Ecoli-5s. Among the data of some 1-min measurements, a prominently high value was recorded at the beginning. The reason for this phenomenon is unknown, but it might be due to the quirks of sensitive systems, including photomultiplier tubes.

As shown in Fig. 3 and Table 2, bioluminescence intensity of Ecoli-1s and Ecoli-1n are significantly higher than those of Ecoli-0s and Ecoli-0n, respectively. This result suggests that the detection limit of this work is 1.75 × 10^2^ cells (g soil)^−1^ or better.

## Discussion

Positive correlations were clearly indicated between the density of *E. coli* and the bioluminescence in both samples with and without the Mars soil simulant. In contrast, it is not trivial to explain the difference of intensity of bioluminescence and its change over time between samples with and without the Mars soil simulant. Simple optical turbidity by the soil remnant particle, which is just a physical effect, can reduce the measured bioluminescence. However, such simple turbidity moves the plots in Fig. 3 downward in parallel. So this effect cannot be the reason why bioluminescence is basically constant in samples with the soil simulant and is exponentially decay in samples with no simulant. A possible hypothesis is that the soil simulant suppresses the rate of biochemical reactions; in general, a microorganism adsorbed well to a soil particle, and therefore flux might become smaller and the reaction might be suppressed.

Schulze-Makuch et al. [5] analyzed soil sample of the Atacama Desert using various methods including ATP measurement in which centrifuge was applied. Centrifuge is effective to remove sand component in the sample, which is unwanted for the measurement of bioluminescence. However, a centrifuge machine is large and heavy in general. This fact is a disadvantage of a centrifuge when we try to apply it for planetary exploration missions. On the other hand, the method applied for Ecoli-0s–Ecoli-5s in this work does not need centrifuge, and therefore, procedures and hardware for it is simple. This fact is an advantage to apply the method for planetary exploration mission. Filtration has potential to be realized by smaller instrument than centrifuge. It is important for future works to design hardware for a planetary exploration and develop an optimal instrument for ATP measurement.

As described in the section of Introduction, it is not guaranteed that extraterrestrial life uses ATP. Therefore, ATP measurement should be performed in extraterrestrial life search with other complimentary methods. If an extraterrestrial life uses ATP, there are two possible scenarios: one is that the extraterrestrial life occurred and evolved independently from terrestrial life, and the other is that an ancestors moved interplanetary. Indeed, interplanetary or interstellar move of life is discussed as panspermia hypothesis especially in the field of astrobiology [13, 14]. ATP measurement is important to examine these scenarios even if the result is negative (i.e., no detection of ATP).

ATP measurement is useful for both in-situ experiment in planetary explorations and analysis for returned samples by planetary explorations. In the case of terrestrial life, ATP is produced well by an active life, and tend to be decomposed easily. So, in extraterrestrial life search, ATP measurement is especially suitable for in-situ experiment of fresh samples obtained by an instrument carried on a spacecraft (e.g., a planetary lander/rover). In contrast, application to returned samples is also useful when it is used with consideration for the possibility of decomposing of ATP in long time of the return way. ATP measurement is applicable not only to Mars sample but also to sample of other place, e.g., icy bodies like Galilean satellites of Jupiter and Saturn, Venus cloud, and asteroids. Among issues relating astrobiology, ATP measurement is also very useful for planetary protection in which contamination from the earth to other planets and that of opposite direction are essential issue. Moreover, it is possible to apply ATP measurement for research of microorganisms in extreme environments such as the Atacama Desert and Antarctica.

A fluorescence microscope is considered to be one of the promising tool for search and analysis for extraterrestrial life [16–18], in which targeted sensitivity limit is ~ 10^4^ cells g^−1^ soil. This target is set based on studies for microorganism density of Atacama Desert where is considered as one of the place where microorganism density is the lowest on the earth [19–23]. Thus, the result of this work suggests that its sensitivity can be higher than the sensitivity of fluorescence microscope. A microscope brings us spatial information like morphology, size, and color gradient, but ATP measurement cannot provide spatial information at all. On the other hand, ATP measurement is applicable to microorganisms hidden in small space like crack of soil particle, for which microscope is difficult to apply. Therefore, these two methods are complimentary.

Whilst ATP is a fragile material, adenosine diphosphate (ADP) and AMP are materials produced after the decomposition of ATP, and much more stable than ATP. It is possible to detect these materials with a similar method using bioluminescence as done in this work for ATP. Combination of measurements for ATP, ADP, and AMP can provide more detailed information.
